# Gene Expression Patterns of Dengue Virus-Infected Children from Nicaragua Reveal a Distinct Signature of Increased Metabolism

**DOI:** 10.1371/journal.pntd.0000710

**Published:** 2010-06-15

**Authors:** P'ng Loke, Samantha N. Hammond, Jacqueline M. Leung, Charles C. Kim, Sajeev Batra, Crisanta Rocha, Angel Balmaseda, Eva Harris

**Affiliations:** 1 Department of Medical Parasitology, School of Medicine, New York University, New York, New York, United States of America; 2 Sustainable Sciences Institute, Managua, Nicaragua; 3 Department of Biochemistry and Biophysics, University of California San Francisco, San Francisco, California, United States of America; 4 Unidad de Infectología, Hospital Infantil Manuel Jesús de Rivera, Managua, Nicaragua; 5 Departamento de Virología, Centro Nacional de Diagóstico y Referencia, Ministerio de Salud, Managua, Nicaragua; 6 Division of Infectious Diseases and Vaccinology, School of Public Health, University of California, Berkeley, California, United States of America; Centers for Disease Control and Prevention, United States of America

## Abstract

**Background:**

Infection with dengue viruses (DENV) leads to a spectrum of disease outcomes. The pathophysiology of severe versus non-severe manifestations of DENV infection may be driven by host responses, which could be reflected in the transcriptional profiles of peripheral blood immune cells.

**Methodology/Principal Findings:**

We conducted genome-wide microarray analysis of whole blood RNA from 34 DENV-infected children in Nicaragua collected on days 3–6 of illness, with different disease manifestations. Gene expression analysis identified genes that are differentially regulated between clinical subgroups. The most striking transcriptional differences were observed between dengue patients with and without shock, especially in the expression of mitochondrial ribosomal proteins associated with protein biosynthesis. In the dengue hemorrhagic fever patients, one subset of differentially expressed genes encode neutrophil-derived anti-microbial peptides associated with innate immunity. By performing a meta-analysis of our dataset in conjunction with previously published datasets, we confirmed that DENV infection *in vivo* is associated with large changes to protein and nucleic acid metabolism. Additionally, whereas *in vitro* infection leads to an increased interferon signature, this was not consistently observed from *in vivo* patient samples, suggesting that the interferon response *in vivo* is relatively transient and was no longer observed by days 3–6 of illness.

**Conclusions/Significance:**

These data highlight important differences between different manifestations of severity during DENV infection as well as identify some commonalities. Compilation of larger datasets in the future across multiple studies, as we have initiated in this report, may well lead to better prediction of disease manifestation via a systems biology approach.

## Author Summary

Dengue is a widespread viral disease for which over 3 billion people are at risk. There are no drug treatments or vaccines available for this disease. It is also difficult for physicians to predict which patients are at highest risk for the severe manifestations known as dengue hemorrhagic fever (DHF) and dengue shock syndrome (DSS). We used genome-wide transcriptional profiling analysis to study peripheral blood responses to dengue among patients from Nicaragua. We found that patients with severe manifestations involving shock had very different transcriptional profiles from dengue patients with mild and moderate illness. We then compared our results with other microarray experiments on dengue patients available from public databases and confirmed that dengue is often associated with large changes to the metabolic processes within cells. This approach could identify prognostic markers for severe dengue as well as provide a better understanding of the pathophysiology associated with different grades of disease severity.

## Introduction

Infection by the four serotypes of dengue virus (DENV 1–4) can lead to outcomes ranging from asymptomatic infection to hemorrhagic fever and shock [1]. While many epidemiological risk factors have been identified for the development of the severe forms of disease [2], dengue hemorrhagic fever (DHF) and dengue shock syndrome (DSS), there is still much uncertainty as to the molecular mechanisms underlying pathogenesis [1], [3], [4]. Identifying signatures of host genome-wide transcriptional patterns can be a tool for biomarker discovery as well as for understanding molecular mechanisms and pathophysiological signatures of disease states. This approach has been applied in cancer biology [5] and infectious diseases [6], [7]. Recently, there have been several reports of expression profiling studies in DENV-infected patients [8], [9], [10], [11] and macaques [12]. Other groups have monitored gene expression in cell lines and primary cells infected with DENV *in vitro* [13], [14], [15]. By integrating results from *in vitro* studies as well as multiple *in vivo* studies from different field sites, it may be possible to assemble a comprehensive picture of DENV-host interactions that lead to different disease outcomes.

The four DENV serotypes have been reported to exhibit distinct clinical characteristics [16], [17], [18]. It is currently unknown whether different serotypes are associated with distinct transcriptional profiles from peripheral blood. Unlike Southeast Asia where all DENV serotypes circulate simultaneously, at our study site in Nicaragua, one serotype tends to predominate each dengue season [18]. In 2003, when this study was conducted, 87% of the isolated viruses were DENV-1 serotype. To our knowledge, this is the first reported transcriptional profiling study of predominantly DENV-1-infected patients in the Americas rather than Southeast Asia.

The goal of our study was to conduct a transcriptional profiling analysis of pediatric patients from Nicaragua with a predominantly DENV-1 infection and to compare our results with Southeast Asian studies with mixed serotype infections. We find that there is a distinct molecular profile between Nicaraguan dengue patients with shock relative to patients with DHF, and a more muted difference between DF patients and patients with DHF. We also conducted a microarray ‘meta-analysis’ to compare the dataset from Nicaraguan patients with existing dengue expression profiling datasets to identify common themes and differences.

## Materials and Methods

### Study design and clinical definitions

A cross-sectional study was conducted in the Hospital Infantil Manuel de Jesús Rivera (HIMJR), the national pediatric reference hospital, in the capital city of Managua, Nicaragua, from September 2003 to February 2004, which represents the 2003 dengue season (see Supplemental Methods). Enrollment criteria consisted of hospitalized patients younger than 15 years of age who completed the informed consent and assent process and who presented with acute febrile illness and two or more of the following symptoms: headache, retro-orbital pain, myalgia, arthralgia, rash, and hemorrhagic manifestations. Subjects reflected the gender, age, and ethnic composition of the local pediatric population. A standardized questionnaire was administered to collect demographic and clinical information at admission, and clinical data during hospitalization was prospectively collected using standardized forms that were completed and verified via chart review. Venous blood was drawn for clinical tests and serological, virological and molecular biological dengue diagnostic assays when patients presented to the Infectious Diseases Unit, daily during hospitalization, at the time of discharge, and when possible approximately two weeks after symptom onset (convalescent sample). Venous blood was collected in PAXgene tubes for RNA analysis at presentation, every other day during hospitalization, and when possible at convalescence. We selected samples from 34 patients for microarray analysis who presented between days 3-6 of illness. Patients were classified according to the 1997 World Health Organization classification scheme as dengue fever (DF), DHF, or DSS [19].

### Ethics statement

This study was conducted according to the principles expressed in the Declaration of Helsinki. Written informed consent was obtained from parents or legal representatives authorizing the participation of their children in the study and assent was obtained from children greater than 5 years old. This study was approved by the University of California, Berkeley Committee for the Protection of Human Subjects, the Ethical Review Committee of the Centro Nacional de Diagnóstico y Referencia (CNDR) of the Nicaraguan Ministry of Health, and the Institutional Review Board of the HIMJR.

### Microarray processing and data analysis

Samples were drawn into PAXgene tubes (BD, Franklin Lakes, NJ) to maintain RNA integrity and provide a snapshot of *in vivo* blood expression while minimizing time and manipulation variations (see Supplemental Methods). RNA was then amplified using the Amino Allyl MessageAmp II aRNA Amplification Kit (Ambion). A reference consisting of an equal quantity of amplified RNA from a pool of RNA extracted from PBMCs of 5 healthy individuals was labeled with Cy3 dye and hybridized against the Cy5-labeled controls on Human Exonic Evidence-Based Oligonucleotide (HEEBO, Invitrogen) microarrays printed in-house at the UCSF Center for Advanced Technologies. This oligonucleotide set (44,544 70mer probes) was designed using a transcriptome-based annotation of exonic structure for genomic loci (http://www.microarray.org/sfgf/heebo.do). Typically one microarray was performed for each patient sample and the quality of the image was checked by using the HEEBO quality assessment graphs using the Bioconductor package. Microarrays that were of poor quality (high background, low foreground) were repeated. Two main strategies were used to identify related subgroups of transcripts and patient subgroups. First, we used unsupervised approaches such as hierarchical clustering and principal component analysis (PCA) for classification of transcripts and patients; this does not utilize clinical information from the patients, and sub-groups are identified based purely on transcriptional profiling data (see Supplemental Methods). Second, patients were divided into subgroups (DF, DHF, and DSS) based exclusively on clinical information, and gene expression analysis was focused on identifying groups of genes that were differentially expressed significantly between pre-defined clinical groups (see Supplemental Methods). This type of supervised comparison was used to identify genes that were significantly up-regulated in DF, DHF and DSS patient samples relative to convalescent patient samples. This supervised approach was also used to identify candidate genes over-represented in patient groups by multi-class SAM analysis (see below).

### Quantitative qRT-PCR

Reverse transcription was performed with 100 ng of the RNA samples from the 34 individuals and Superscript III (Invitrogen). Quantitative RT-PCR (qRT-PCR) of the samples was performed in triplicate using Taqman probes (Defensin alpha 1 (Hs00234383_m1), Lactotransferrin (Hs00158924_m1), CD14 (Hs00169122_g1), PRAM1 (Hs00259796_m1), vezatin (Hs00250257_m1), ZNF600 (Hs01650984_g1), ZNF91 (Hs00602754_mH), EIF-1a (Hs00796778_s1)) and kit (Applied Biosystems) according to the manufacturer's instructions on a Step One Plus real-time PCR system (Applied Biosystems). Samples with cycle thresholds (Ct) values of >36 cycles for a given probe were considered undetectable (ud). Ct values were then normalized to the value of a housekeeping gene (HPRT), and data is presented as a ratio to HPRT.

### Meta-analyses of dengue expression profiling datasets

For studies with corresponding Genepix or Affymetrix CEL files available in a public repository, the original files were downloaded and processed using a standardized workflow (see Supplemental Methods). For studies that did not have original intact array files available, we relied on the authors' preprocessed data. Expression values were log_2_ transformed and arrays were median centered. Groups of arrays within each study were collapsed by averaging, and all of the arrays were also “zero”-transformed by a set of healthy/convalescent samples (for *in vivo* studies) or uninfected/heat-inactivated virus-treated samples (for *in vitro* studies) within each study. Data was then mapped to Human HGNC gene ID's (see Supplemental Methods). Finally, all the columns in the integrated dataset were standardized (z-score transformed).

### Pathway analysis/statistical methods

Filtered datasets were also analyzed for statistically significant genes using the Statistical Analysis of Microarray (SAM) software version 2.23A [20]. SAM was used for supervised two-way comparisons as well as multi-class analysis. Genes identified by clustering or SAM analysis were visualized using the Treeview (http://rana.lbl.gov/EisenSoftware.htm). Gene ontology and pathway analyses were performed using PANTHER (http://www.pantherdb.org/) [21]. Gene lists were introduced into PANTHER along with a background gene list representing all of the genes present in the human genome. Statistical comparisons of qRT-PCR data by the non-parametric two-tailed Mann-Whitney test was conducted using PRISM (Graphpad).

## Results

### Research subject characteristics

We analyzed samples collected from confirmed DENV-infected individuals in Nicaragua recruited during the dengue season of 2003, with predominantly DENV-1 in circulation [17]. PAXgene tubes were analyzed from 34 individuals with samples collected between days 3–6 of illness on the day of admission (Table 1). All of the individuals were hospitalized in the Infectious Disease Unit of the Hospital Infantil Manuel de Jesús Rivera (HIMJR). Twenty patients were classified as dengue fever (DF) according to the traditional WHO criteria [19]. Six patients developed DHF, and eight patients developed DSS (Table 1). Convalescent samples from six patients (two with DF, one with DHF, three with DSS) were also collected for analysis 14–19 days after onset of symptoms, after they were discharged from the hospital.

**Table 1:** Clinical features of confirmed dengue cases by disease classification.

Variable	DF (n = 20) Mean (range)	DHF (n = 6) Mean (range)	DSS (n = 8) Mean (range)
**Age, yrs**	7 (9m-12)	12 (7–14)	7 (10m-14)
**Platelet nadir, cells/ml**	77,600 (18,000–148,000)	60,000 (39,000–81,000)	49,000 (24,000–65,000)
**Leucocytes nadir, cells/ml**	4,065 (1,500–9,100)	3,683 (1,900–6,500)	4,337 (1,800–8,700)
**Hct, nadir, %**	33.3 (25–39)	34.5 (30–38)	31.8 (26–37)
**Hct peak, %**	37.6 (33–47)	40.2 (36–43)	36.6 (27–45)
**Hct discharge, %**	35.6 (29–44)	36.7 (30–42)	33.4 (27–40)
**Days of illness at time of sample collection**	4.2 (3–6)	4.2 (3–5)	4.6 (3–6)

Variable	n (%)	n (%)	n (%)
**Gender male**	10 (50)	3 (50)	7 (88)
**Primary infection**	4 (20)	2 (33)	2 (25)
**Secondary infection**	16 (80)	4 (67)	6 (75)
**Thrombocytopenia (<100,000 cells/ml)**	16 (80)	6 (100)	8 (100)
**Plasma leakage** *	3 (15)	6 (100)	6 (75)
**Narrow pulse pressure**	0	0	3 (38)
**Hypotension**	0	0	5 (63)
**Slow capillary refill (>2 sec)**	1 (5)	2 (33)	3 (38)
**Cold clammy skin**	3 (15)	3 (50)	5 (63)

*Elevated hematocrit for age or when compared to discharge Hct or other signs of plasma leakage (pleural effusion, ascites).

### Overall gene expression profiles correlate with clinical status

**Figure 1:**
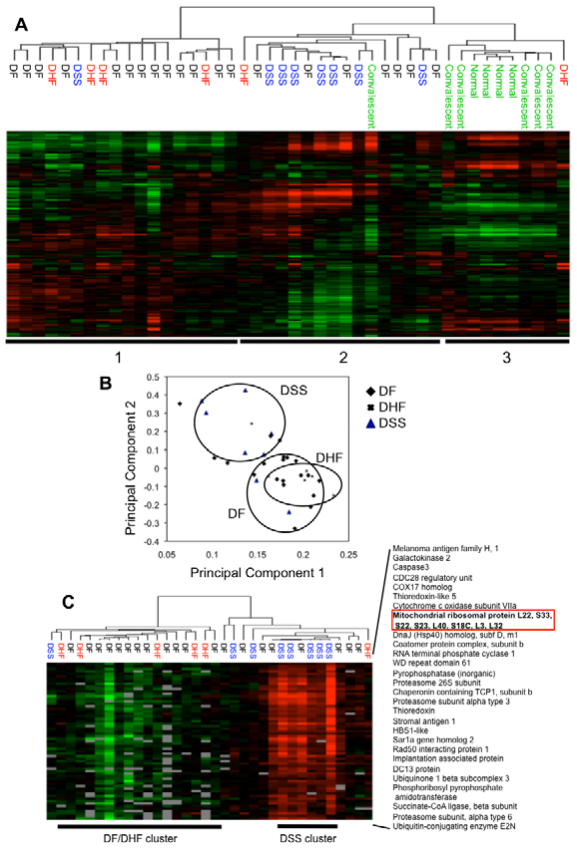
Unsupervised hierarchical clustering analysis of peripheral blood expression profiles from DENV-infected individuals. (A) Gene expression patterns of peripheral blood samples from DENV-infected patients during acute illness (3–6 days) and convalescence as well as healthy individuals. Clustering was used to organize genes and samples. Each row represents an individual gene and each column an individual patient. Black indicates the median level of expression, red indicates greater than median expression, green depicts less than median expression, and gray indicates missing data. Horizontal bars (labeled 1, 2 and 3) indicate the grouping of mostly DF/DHF patients, DSS patients, and healthy/convalescent individuals,respectively. (B) Principal Component Analysis (PCA) of transcriptional profiling data. Each dot represents a DENV-infected individual plotted in two dimensions using their projections onto the first two principal components. Samples are coded according to clinical categories of DF, DHF, and DSS. The circles represent clusters containing the majority (>75%) of DSS, DF and DHF patients. (C) Reorganization of samples based on expression values of 61 genes identified to contribute towards the top 5% of positive variance explained by PCA2 in the Principal Component Analysis of the dataset. Horizontal bars indicate a cluster of DSS patients and a cluster of DF/DHF patients.

We used hierarchical clustering to determine whether the molecular profiles alone could distinguish between patients with different clinical manifestations. The unsupervised organization of the samples based on gene expression levels of all available genes post-filtering (n = 8,485) led to the segregation into primarily three groups (Figure 1A). The first group (horizontal bar 1) contained most of the DHF patients (4/6, with one patient closely associated) and the majority of the DF patients (13/20). The second group (horizontal bar 2) included almost all (7/8) of the DSS patients. The third group (horizontal bar 3) included all (4/4) of the healthy controls and most (5/6) of the convalescent samples.

We then applied Principal Component Analysis (PCA) to identify internal structures within the dataset. The variance explained by the principal components when PCA is applied is shown in Figure S1. The first two components (PCA1 and PCA2) explain much more variance than the other components. We then projected each patient sample onto these two components (Figure 1B) and observed that the majority (>75%) of the DSS patients clustered together, but were separate from the majority (>75%) of DF and DHF patients. We also observed that the values for PCA2 appeared to separate the DSS samples from the DF and DHF samples more distinctly than PCA1, and this was statistically significant (Figure S1B). We then identified 61 genes (from 66 data spots) that contributed towards the top 5% of the positive variance explained by PCA2 (Figure S1C). When we reclustered the patient samples based on the expression patterns of these 61 genes, we found that almost all of the DSS patients clustered together (7/8), while the majority of the DF and DHF patients also clustered together (Figure 1C). We also identified the top 5% of genes (n = 90, from 93 spots) that were positively correlated with PCA2 (n = 1832 at a false discovery rate of 5%), and reclustering with these 90 genes produced a similar result (Figure S1D).

To identify which biological processes were over-represented in the set of 61 genes, we performed gene ontology (GO) analysis. Most of the genes that were associated with shock appear unrelated to the immune response, while genes involved in ‘protein biosynthesis’ (n = 11) and ‘protein metabolism and modification’ (n = 21) were significantly enriched (P<0.05) in this list (Table S1). A large number of mitochondrial ribosomal proteins are also found on this list, reflecting an enrichment of ‘ribosomal proteins’ (n = 9, P = 2.7E-04) and of ‘nucleic acid binding’ (n = 16, P = 2.56E-02) molecular functions. GO analysis of the 90 genes representing the top 5% of genes correlated with PCA2 also produced similar results (Figure S1E).

### Identifying differentially expressed genes through supervised comparisons

**Figure 2:**
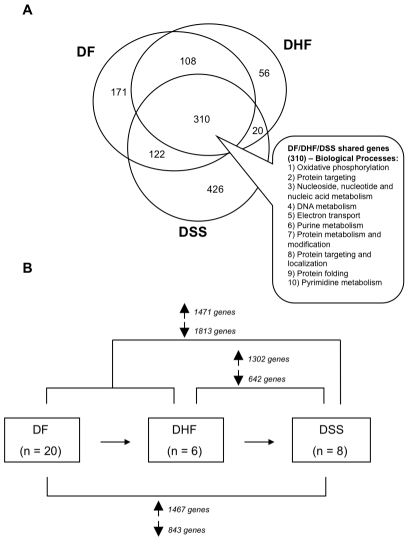
Supervised comparisons between expression values of pre-defined patient categories. (A) Venn diagram identifying genes (n = 310) that are up-regulated in common between all categories of DENV-infected patients relative to convalescent samples. Up-regulated genes were identified through two-way SAM comparisons between each group (DF, n = 20; DHF, n = 6; DSS, n = 8) and convalescent samples (n = 6). GO analysis was used to identify “biological processes” significantly over-represented in this list of “common” genes from all DENV-infected patients. (B) Multiple two-way SAM comparisons between patients of different clinical categories showing the numbers of significantly up or down-regulated genes in each comparison at a false discovery rate of 5%. Genes identified through these two-way comparisons are listed in Table S5.

We next conducted supervised two-way comparisons using the statistical analysis of microarrays (SAM) method [20] to compare expression of transcripts between the acute (DF: n = 20; DHF: n = 6; DSS: n = 8) and convalescent samples (n = 6). Although there were not sufficient convalescent samples to make autologous comparisons, we identified 964 genes that were differentially expressed (711 up-regulated, 253 down-regulated) in acute DF samples compared to all of the convalescent samples (Table S2), 550 genes that were differentially expressed (494 up-regulated, 56 down-regulated) in acute DHF acute samples (Table S3) and 1203 genes that were differentially expressed (878 up-regulated and 325 down-regulated) in DSS acute samples (Figure 2A, Table S4). A large number of genes that were up-regulated during acute illness relative to convalescence (n = 310) are shared between all the groups of dengue patients (Figure 2A). This could represent a “common” response to DENV infection, regardless of the final clinical outcome of the patients. Surprisingly, most of these genes appear unrelated to the immune response and instead belong mainly to metabolism-related processes (oxidative phosphorylation, protein targeting, nucleic acid metabolism, purine and pyrimidine metabolism, electron transport, DNA metabolism and replication, and protein metabolism and modification) through GO analysis (Figure 2A).

We then conducted multiple two-way SAM comparisons between patients of different clinical classifications at a 5% false discovery rate (Figure 2B and Table S5). There are large differences between the acute samples taken from DF and DHF patients relative to the DSS patients (Figure 2B). Using GO analysis, the biological processes enriched in genes differentially expressed by DSS patients were found to include protein metabolism and modification, intracellular protein traffic, pre-mRNA processing, mRNA splicing, nuclear transport, protein-lipid modification and protein folding (Table S6 and Table S7).

While we did not identify any genes (at a 5% FDR) that were significantly different between DF and DHF patients, a group of neutrophil derived anti-microbial peptides (lactotransferrin, defensins alpha 1,3 and 4 and cathelicidin) were among the most highly ranked (data not shown). When we performed a sub-analysis by grouping DF patients with severe thrombocytopenia (platelet count of less than 50,000/mm^3^; “DF+”) [[22] with DHF patients, these genes were identified to be significantly upregulated in the DF+/DHF patients as compared to uncomplicated DF.

As an additional strategy to determine if we could identify candidate classification genes for different dengue patient categories, we used multi-class SAM analysis to identify transcripts that are up- or down-regulated among the different patient categories (DF, DHF and DSS). At a FDR rate of less than 5%, we identified 1525 transcripts that were differentially expressed between the different patient categories (Table 2), including inflammatory markers (lymphotoxin beta receptor, PRAM1, CD14) that were more highly expressed in the DHF group.

**Table 2:** Most differentially expressed genes between patient categories.

Gene Name	DF	DHF	DSS
ORM1-like 1 (S. cerevisiae)	−0.53	−1.82	2.69
transmembrane protein vezatin	−0.41	−1.86	2.42
dynactin 6	−0.60	−1.41	2.54
high mobility group nucleosomal binding domain 3	−0.71	−1.01	2.52
proteasome (prosome, macropain) subunit, alpha type, 4	−0.75	−0.53	2.28
zinc finger protein 292	−0.63	−1.29	2.54
THUMP domain containing 2	−0.71	−0.82	2.40
similar to ribosomal protein L22	−0.66	−0.73	2.19
PTX1 protein	−0.57	−1.14	2.29
LSM8 homolog, U6 small nuclear RNA associated (S. cerevisia	−0.65	−0.78	2.20
likely ortholog of mouse immediate early response, erythrop	−0.67	−0.84	2.30
chromosome 14 open reading frame 112	−0.71	−0.62	2.23
histone acetyltransferase 1	−0.66	−0.70	2.17
NA	−0.62	−1.00	2.30
HDCMA18P protein	−0.68	−0.77	2.27
SUMO-1 activating enzyme subunit 2	−0.53	−1.20	2.23
small nuclear ribonucleoprotein polypeptide B″	−0.58	−1.27	2.40
PRAM-1 protein	0.44	1.41	−2.16
HCV NS3-transactivated protein 1	−0.67	−0.75	2.24
immediate early response 3 interacting protein 1	−0.55	−1.03	2.16
transmembrane protein 14B	−0.44	−1.66	2.33
PTX1 protein	−0.51	−1.19	2.16
C-type lectin superfamily 2, member D	−0.57	−0.95	2.13
mitochondrial ribosomal protein S18C	−0.70	−0.53	2.14
lymphotoxin beta receptor (TNFR superfamily, member 3)	0.22	1.88	−1.95
RPA interacting protein	−0.70	−1.09	2.58
small nuclear ribonucleoprotein polypeptide G	−0.66	−0.63	2.12
CD14 antigen	0.23	1.85	−1.96
RNA (guanine-9-) methyltransferase domain containing 1	−0.57	−1.08	2.23
gene model 83	−0.60	−0.92	2.19
LSM3 homolog, U6 small nuclear RNA associated (S. cerevisia	−0.62	−0.78	2.13
myoneurin	−0.63	−0.79	2.15
G-rich RNA sequence binding factor 1	−0.71	−0.86	2.41
cystatin C (amyloid angiopathy and cerebral hemorrhage)	0.21	1.91	−1.96
TatD DNase domain containing 1	−0.62	−0.78	2.13
zinc finger, ZZ domain containing 3	−0.62	−0.80	2.14
polymerase (RNA) II (DNA directed) polypeptide K, 7.0kDa	−0.43	−1.47	2.17
KIAA0020	−0.67	−0.94	2.39
CGI-12 protein	−0.65	−0.90	2.30
thyroid hormone receptor interactor 3	−0.66	−0.65	2.12
NudC domain containing 2	−0.46	−1.21	2.05
Tax1 (human T-cell leukemia virus type I) binding protein 1	−0.49	−1.30	2.20
programmed cell death 10	−0.64	−0.58	2.03
TatD DNase domain containing 1	−0.60	−0.80	2.11
KIAA0776	−0.43	−1.20	1.98
NADH dehydrogenase (ubiquinone) 1 beta subcomplex, 3, 12kDa	−0.73	−0.22	1.99
dolichyl-phosphate mannosyltransferase polypeptide 1, catal	−0.68	−0.49	2.07
similar to ribosomal protein L22	−0.69	−0.29	1.94
cyclin C	−0.35	−1.36	1.90
translocase of inner mitochondrial membrane 17 homolog A (y	−0.55	−0.99	2.11
ubiquitin carboxyl-terminal esterase L3 (ubiquitin thiolest	−0.63	−0.90	2.25
transposon-derived Buster1 transposase-like protein gene	−0.61	−0.81	2.13
heat shock 10kDa protein 1 (chaperonin 10)	−0.48	−1.17	2.08
eukaryotic translation initiation factor 3, subunit 6 48kDa	−0.33	−1.65	2.06
proteasome (prosome, macropain) 26S subunit, ATPase, 6	−0.60	−0.69	2.03
similar to bA486O22.3 (similar to RPS3A (ribosomal protein	−0.58	−0.82	2.07
proteasome (prosome, macropain) subunit, alpha type, 3	−0.59	−0.80	2.08
baculoviral IAP repeat-containing 2	−0.61	−0.76	2.10
chondroitin sulfate proteoglycan 6 (bamacan)	−0.56	−0.98	2.13
KIAA1040 protein	−0.64	−0.74	2.15
Rab geranylgeranyltransferase, beta subunit	−0.41	−1.33	2.02
dolichyl-phosphate mannosyltransferase polypeptide 1, catal	−0.61	−0.73	2.08
solute carrier family 43, member 2	0.46	1.25	−2.09
mitochondrial ribosomal protein L32	−0.61	−0.75	2.09
caspase recruitment domain family, member 9	0.15	2.07	−1.92
ATP-binding cassette, sub-family E (OABP), member 1	−0.49	−1.11	2.07
CD68 antigen	0.04	2.07	−1.65
tubulin-specific chaperone a	−0.50	−1.14	2.10
EPM2A (laforin) interacting protein 1	−0.05	−2.29	1.84
nucleolar protein 8	−0.52	−1.34	2.30
kinectin 1 (kinesin receptor)	−0.52	−1.10	2.13
activated RNA polymerase II transcription cofactor 4	−0.49	−1.00	1.96
COP9 constitutive photomorphogenic homolog subunit 4 (Arabi	−0.59	−0.91	2.16
heat shock 10kDa protein 1 (chaperonin 10)	−0.56	−0.93	2.09
chromosome 21 open reading frame 66	−0.61	−0.87	2.18
apolipoprotein B48 receptor	0.35	1.48	−1.99
peptidyl-prolyl isomerase G (cyclophilin G)	−0.59	−0.88	2.13
pinin, desmosome associated protein	−0.66	−0.66	2.14
heat shock 70kDa protein 14	−0.59	−0.78	2.06
jumonji domain containing 3	0.26	1.72	−1.95
hemoglobin, alpha 1	0.30	1.46	−1.83
hypothetical protein MGC5509	−0.52	−1.11	2.13
mitochondrial ribosomal protein L47	−0.64	−0.57	2.02
mitochondrial ribosomal protein L1	−0.57	−0.81	2.02
cylindromatosis (turban tumor syndrome)	−0.80	−0.44	2.33
stress-associated endoplasmic reticulum protein 1	−0.48	−1.10	2.03
lectin, galactoside-binding, soluble, 9 (galectin 9)	0.14	2.09	−1.91
zinc finger, DHHC domain containing 17	−0.56	−0.92	2.10
hypothetical protein DKFZp586C1924	−0.67	−0.51	2.06
dolichyl-phosphate mannosyltransferase polypeptide 1, catal	−0.60	−0.65	2.00
proteasome (prosome, macropain) subunit, alpha type, 2	−0.67	−0.65	2.17
mitochondrial ribosomal protein L13	−0.49	−1.00	1.98
DnaJ (Hsp40) homolog, subfamily A, member 1	−0.84	−0.19	2.23
TGF beta-inducible nuclear protein 1	−0.69	−0.59	2.17
solute carrier family 35 (CMP-sialic acid transporter), mem	−0.61	−0.85	2.17
Sjogren syndrome antigen B (autoantigen La)	−0.76	−0.37	2.18
source of immunodominant MHC-associated peptides	−0.47	−1.06	1.97
metaxin 3	−0.51	−1.06	2.07
likely ortholog of mouse hypoxia induced gene 1	−0.58	−0.76	2.03
thioredoxin domain containing 10	−0.55	−0.86	2.02

Values shown represent the contrast value for each gene. This is the standardized mean difference between the gene's expression in that class, versus its overall mean expression.

**Figure 3:**
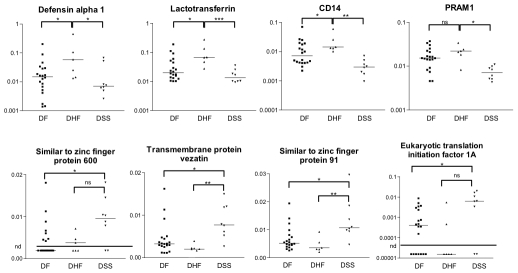
Verification of gene expression by real-time PCR analysis of DENV-infected patient peripheral blood samples. Transcript levels of selected genes were measured and normalized to b-actin transcript levels. Horizontal bars indicate median expression levels. Statistical significance between groups were determined by the 2-tailed Mann-Whitney test. A *p* value of <0.05 is indicated by *; *p*<0.01 is indicated by **, and *p*<0.001 is indicated by ***. In some cases, transcripts were below the detection limit, as indicated by the horizontal bar (nd).

We then used TaqMan real-time PCR primers to validate a subset of candidate classification genes. including the group of neutrophil-derived anti-microbial peptides that may be correlated with DHF responses (Figure 3) We found that expression of lactotransferrin and defensin alpha 1/3 was significantly higher in DHF patients relative to both DF and DSS patients. PRAM1 is down-regulated in DSS patients relative to both DHF and DF patients, as is CD14. We also verified the expression of some transcripts that were associated with DSS. The transmembrane protein vezatin and the transcription factor similar to zinc finger protein 91 were significantly up-regulated in DSS patients relative to both DF as well as DHF patients. The transcription factor similar to zinc finger protein 600 and the eukaryotic translation initiation factor 1A were also significantly up-regulated in DSS patients relative to DF patients, but not significantly different from DHF patients.

### Meta-analyses of dengue expression profiling studies

Next, we compared our results with two *in vitro* [13], [14] and two other *in vivo* studies [8], [9], [10] for which expression datasets are publicly available. We searched for a “common dengue response” from all *in vitro* and *in vivo* studies (Table 3 and Table S8). The biological processes that were over-represented in the up-regulated genes were DNA replication, cell-cycle and DNA metabolism, suggesting that DENV infection leads to an increase in the cell cycle machinery, DNA replication and the nucleic acid metabolism pathways necessary to support these processes. In contrast, signal transduction pathways may be suppressed during DENV infection. The immunity and defense process as well as the blood-clotting process were also over-represented in the down-regulated gene list.

**Table 3:** Top 100 “Common dengue response” up-regulated genes identified through a meta-analysis of *in vitro* and *in vivo* transcriptional profiling studies.

Gene ID	Gene Name	Score(d)
3071	(DUSP5) dual specificity phosphatase 5	7.08
1508	(CASP7) caspase 7, apoptosis-related cysteine peptidase	5.92
3527	(EZH2) enhancer of zeste homolog 2 (Drosophila)	5.61
1667	(CD38) CD38 molecule	5.39
20090	(DNAJC1) DnaJ (Hsp40) homolog, subfamily C, member 1	5.36
2000	(CKS2) CDC28 protein kinase regulatory subunit 2	5.33
1132	(BTG3) BTG family, member 3	5.14
20461	(NME7) non-metastatic cells 7, protein expressed in (nucleoside-diphosphate kinase)	5.13
2347	(CREB3) cAMP responsive element binding protein 3	5.09
8907	(PGM3) phosphoglucomutase 3	5.05
12311	(TRIP6) thyroid hormone receptor interactor 6	4.85
10702	(SEC23B) Sec23 homolog B (S. cerevisiae)	4.82
1176	(MRPL49) mitochondrial ribosomal protein L49	4.81
10703	(SEC24A) SEC24 family, member A (S. cerevisiae)	4.71
4162	(GARS) glycyl-tRNA synthetase	4.71
9569	(PSME2) proteasome (prosome, macropain) activator subunit 2 (PA28 beta)	4.71
6948	(MCM5) minichromosome maintenance complex component 5	4.71
17895	(UBE2S) ubiquitin-conjugating enzyme E2S	4.70
21348	(MLF1IP) MLF1 interacting protein	4.69
2718	(DDB2) damage-specific DNA binding protein 2, 48kDa	4.67
1148	(BUB1) budding uninhibited by benzimidazoles 1 homolog (yeast)	4.64
18643	(SF4) splicing factor 4	4.62
20144	(ZNF410) zinc finger protein 410	4.61
3650	(FEN1) flap structure-specific endonuclease 1	4.59
18538	(NUSAP1) nucleolar and spindle associated protein 1	4.59
11302	(SRP68) signal recognition particle 68kDa	4.55
6388	(KIF11) kinesin family member 11	4.48
7397	(MT1E) metallothionein 1E	4.42
1745	(CDC7) cell division cycle 7 homolog (S. cerevisiae)	4.41
1504	(CASP3) caspase 3, apoptosis-related cysteine peptidase	4.41
92	(ACADVL) acyl-Coenzyme A dehydrogenase, very long chain	4.40
10451	(RRM1) ribonucleotide reductase M1	4.37
17943	(DERL2) Der1-like domain family, member 2	4.32
7398	(MT1F) metallothionein 1F	4.31
17598	(UBE2J1) ubiquitin-conjugating enzyme E2, J1 (UBC6 homolog, yeast)	4.31
6395	(KPNA2) karyopherin alpha 2 (RAG cohort 1, importin alpha 1)	4.31
14938	(PIGT) phosphatidylinositol glycan anchor biosynthesis, class T	4.29
15832	(BSCL2) Bernardinelli-Seip congenital lipodystrophy 2 (seipin)	4.29
17493	(GMNN) geminin, DNA replication inhibitor	4.27
17064	(ELL2) elongation factor, RNA polymerase II, 2	4.26
7693	(NDUFA9) NADH dehydrogenase (ubiquinone) 1 alpha subcomplex, 9, 39kDa	4.25
4716	(HIST1H1C) histone cluster 1, H1c	4.25
10044	(RNASE1) ribonuclease, RNase A family, 1 (pancreatic)	4.25
29608	(MTDH) metadherin	4.24
9226	(PPA1) pyrophosphatase (inorganic) 1	4.21
17981	(RTCD1) RNA terminal phosphate cyclase domain 1	4.21
25009	(UBE2T) ubiquitin-conjugating enzyme E2T (putative)	4.20
1739	(CDC45L) CDC45 cell division cycle 45-like (S. cerevisiae)	4.19
20038	(DDX52) DEAD (Asp-Glu-Ala-Asp) box polypeptide 52	4.18
17357	(APOBEC3G) apolipoprotein B mRNA editing enzyme, catalytic polypeptide-like 3G	4.17
25585	(OGFOD1) 2-oxoglutarate and iron-dependent oxygenase domain containing 1	4.14
19071	(THOC4) THO complex 4	4.11
9972	(RFC4) replication factor C (activator 1) 4, 37kDa	4.10
952	(BARD1) BRCA1 associated RING domain 1	4.09
1082	(BNIP1) BCL2/adenovirus E1B 19kDa interacting protein 1	4.08
11300	(SRP19) signal recognition particle 19kDa	4.08
13266	(DDX24) DEAD (Asp-Glu-Ala-Asp) box polypeptide 24	4.08
949	(BAK1) BCL2-antagonist/killer 1	4.07
17022	(HPS5) Hermansky-Pudlak syndrome 5	4.06
11913	(TNFRSF17) tumor necrosis factor receptor superfamily, member 17	4.03
8729	(PCNA) proliferating cell nuclear antigen	4.02
9316	(PPP3CC) protein phosphatase 3 (formerly 2B), catalytic subunit, gamma isoform	4.02
13870	(NUDT21) nudix (nucleoside diphosphate linked moiety X)-type motif 21	4.00
16870	(MELK) maternal embryonic leucine zipper kinase	3.98
372	(AKAP2) A kinase (PRKA) anchor protein 2	3.98
28351	(C11orf48) chromosome 11 open reading frame 48	3.98
7406	(MT2A) metallothionein 2A	3.96
3245	(EHD4) EH-domain containing 4	3.96
16429	(LIAS) lipoic acid synthetase	3.95
23115	(EAF2) ELL associated factor 2	3.95
5384	(IDH3A) isocitrate dehydrogenase 3 (NAD+) alpha	3.94
13813	(SLC4A1AP) solute carrier family 4 (anion exchanger), member 1, adaptor protein	3.94
16065	(GLRX2) glutaredoxin 2	3.93
20320	(PSPC1) paraspeckle component 1	3.93
6947	(MCM4) minichromosome maintenance complex component 4	3.91
10381	(RPN1) ribophorin I	3.91
1580	(CCNB2) cyclin B2	3.88
1578	(CCNA2) cyclin A2	3.87
9548	(PSMC2) proteasome (prosome, macropain) 26S subunit, ATPase, 2	3.87
4248	(GGH) gamma-glutamyl hydrolase (conjugase, folylpolygammaglutamyl hydrolase)	3.85
9343	(PRCC) papillary renal cell carcinoma (translocation-associated)	3.85
12417	(TUBG1) tubulin, gamma 1	3.85
17557	(LCMT1) leucine carboxyl methyltransferase 1	3.85
7548	(MYBL2) v-myb myeloblastosis viral oncogene homolog (avian)-like 2	3.84
7434	(MTHFD2) methylenetetrahydrofolate dehydrogenase (NADP+ dependent) 2, methenyltetrahydrofolate cyclohydrolase	3.83
12718	(VRK1) vaccinia related kinase 1	3.83
10942	(SLC1A4) solute carrier family 1 (glutamate/neutral amino acid transporter), member 4	3.81
14211	(BLNK) B-cell linker	3.81
19123	(DNAJC9) DnaJ (Hsp40) homolog, subfamily C, member 9	3.79
18967	(EDEM1) ER degradation enhancer, mannosidase alpha-like 1	3.79
5978	(IL15RA) interleukin 15 receptor, alpha	3.78
10706	(SEC24D) SEC24 family, member D (S. cerevisiae)	3.78
6949	(MCM6) minichromosome maintenance complex component 6	3.76
1101	(BRCA2) breast cancer 2, early onset	3.73
6130	(ISG20) interferon stimulated exonuclease gene 20kDa	3.73
9537	(PSMB1) proteasome (prosome, macropain) subunit, beta type, 1	3.72
2993	(DONSON) downstream neighbor of SON	3.72
9532	(PSMA3) proteasome (prosome, macropain) subunit, alpha type, 3	3.71
12719	(VRK2) vaccinia related kinase 2	3.70
9356	(PREB) prolactin regulatory element binding	3.69
10535	(SAR1B) SAR1 homolog B (S. cerevisiae)	3.69

**Figure 4:**
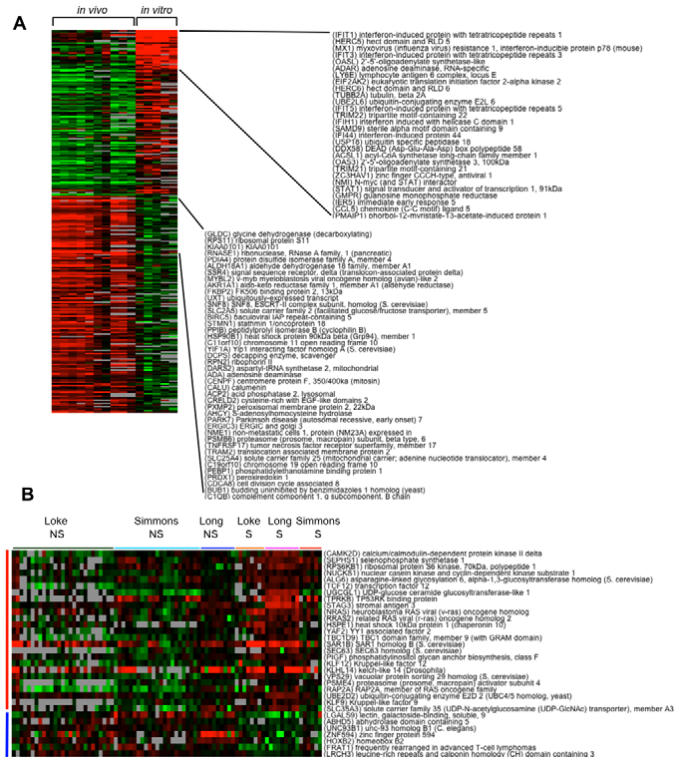
Meta-analyses of integrated DENV expression profiling datasets. (A) Comparison between *in vitro* profiling studies of DENV-infected cells and *in vivo* profiling studies of DENV-infected patients. Each row represents an individual gene and each column an averaged value from the different studies. Black indicates the median level of expression, red indicates greater than median expression, green indicates less than median expression, and gray represents missing data. A subset of genes that are prominently up-regulated in either the *in vitro* or *in vivo* studies are listed. (B) Identification of 32 genes that are significantly different between severe (S; DSS) and non-severe (NS; DF & DHF) dengue cases through a meta-analysis of Vietnamese and Nicaraguan patients. Twenty-five genes were more highly expressed in severe patients (red vertical bar) and 7 genes were more highly expressed in non-severe patients (blue vertical bar). The horizontal bar indicates the respective studies (Loke, Long and Simmons) and classification of the patients as severe (S; DSS) or non-severe (NS; DF&DHF).

We then examined differences between *in vivo* and *in vitro* responses (Figure 4A). With *in vivo* responses, at an FDR of 0%, 1667 genes were differentially expressed, with 943 up-regulated genes and 724 down-regulated genes (Table S9). While many pathways and gene ontology categories were either over- or under-represented, it was interesting to note that many of the “immune signaling” categories were generally under-represented in the up-regulated genes and over-represented in the down-regulated genes, suggesting that DENV infection could be suppressing immune signaling in the host. In contrast, when we examined differentially expressed genes from the *in vitro* studies, we found up-regulated genes that were over-represented in the processes of interferon-mediated immunity, proteolysis, and immunity and defense (Table S10). This interferon signature is not as easily observed in the *in vivo* samples. When we performed a two-way comparison between the *in vitro* and *in vivo* responses (Table S11), the biological processes of “interferon-mediated immunity” and “immunity and defense” were again over-represented in the upregulated genes from the *in vitro* studies, consistent with the observation of an interferon signature in *in vitro* DENV infection, which was stronger than that observed in *in vivo* patient samples. For the 156 genes more highly expressed in the *in vivo* samples, purine metabolism was again over-represented and signal transduction was under-represented, supporting the hypothesis that there is an increase in nucleic acid metabolism and a suppression of signaling in the peripheral blood cells of dengue patients.

Finally, we compared severe versus non-severe dengue cases in the integrated dengue patient dataset, which includes the Nicaraguan patients in our study and the Vietnamese patients in the Simmons and Long studies [9], [10]. At a FDR of 0%, 32 genes were significantly different between the two groups, with 25 genes more highly expressed in severe patients and 7 genes more highly expressed in the non-severe patients (Figure 4B and Table S12). Among the genes up-regulated in severe dengue patients are transcription factors that belong to the Kruppel-like factor family (KLF9 and KLF12), which have mainly been studied in the context of development. Galectin 9, a Tim-3 ligand, is down-regulated in severe dengue patients.

**Figure 5:**
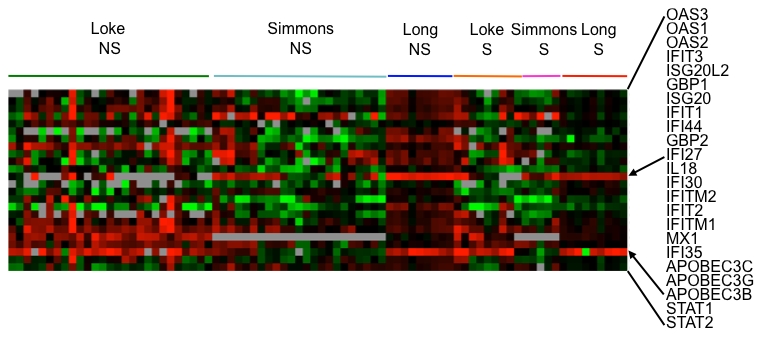
Expression of 24 IFN-upregulated genes in the meta-analysis of dengue patients. Each row represents an individual gene and each column the value from an individual patient from the different studies. Black indicates the median level of expression, red indicates greater than median expression, green indicates less than median expression, and gray represents missing data. The horizontal bar denotes the respective studies (Loke, Long and Simmons) and classification of the patients as severe (S; DSS) or non-severe (NS; DF&DHF).

We were surprised that genes involved in type I interferon production were not more highly represented from the *in vivo* studies, hence we extracted the expression data of 24 genes that are well-characterized as components of the type I IFN signature (Figure 5). While the expression of these genes are clearly up-regulated in DF patients and suppressed in DSS patients as described in the Long et al. study [9], this pattern is less obvious from the Nicaragua dataset, as well as from the Simmons et al. study [10].

## Discussion

In this study, we found that DENV infection *in vivo* is characterized by upregulation of metabolic genes involved in nucleic acid and protein metabolism. We also observed a clear distinction in transcriptional profiles between shock-associated DENV infection relative to infection that led to DHF or DF, characterized mainly by an increased expression of mitochondrial ribosomal proteins. We also found a group of neutrophil-derived anti-microbial peptides (lactotransferrin and defensin A1/A3) up-regulated in DHF patients. Finally, by integrating our dataset with other datasets, we confirmed our observations that genes involved in nucleic acid metabolism are up-regulated during *in vivo* infection. These results suggest that DENV infection leads to large changes in the metabolic pathways of peripheral blood cells.

Why are metabolic pathways upregulated in the peripheral blood during DENV infection *in vivo*? One possibility is that DENV infection induces a strong B cell proliferative response, since there was also an over-representation of immunoglobulin transcripts and protein trafficking categories. Simmons et al. [10] have previously noted an abundance of proliferating B cell plasmablasts that may be associated with the increased expression of genes involved in cell cycle control. Notably, IRF4 and BLNK expression are strongly represented in the *in vivo* meta-analysis, supporting the hypothesis that B cell stimulation, proliferation and differentiation may be a feature of acute DENV infection. It is possible that this signature is further amplified during severe manifestations of dengue, since many genes involved in protein biosynthesis (such as the ribosomal proteins) are further up-regulated, which could be a result of increased antibody production. Another possibility could be the activation of an integrated stress response. It was previously found that expression signatures related to the endoplasmic reticulum (ER) are elevated during acute DENV infection, suggesting the induction of ER stress [10]. The results from both the Nicaragua dataset, as well as our meta-analysis of the integrated dataset, support this hypothesis. Activation of this integrated stress response has also been noted through a systems biology analysis of the yellow fever vaccine [23], as well as in Japanese encephalitis virus- and DENV-2-infected cells [24].

The greater difference observed between DSS versus DF and DHF patients supports the concept of dengue as a spectrum of disease severity, with severe disease characterized by shock [Dengue guidelines for diagnosis, treatment, prevention and control. Third Edition 2009. WHO, Geneva]. The most dramatic changes that occur during severe dengue with shock appear to be effects on cellular physiology rather than on immune responses. Increased expression of mitochondrial ribosomal proteins may be an indication that protein biosynthesis could be ramped up in DSS patients. Understanding why protein biosynthesis may be increased in DSS patients requires further studies. Increased viral replication and viral load [25] and slower rates of viral clearance [26] have been associated with severe manifestations of dengue. It is possible that the increased signature of protein biosynthesis is related to increased production of viral proteins; however, DENV viral load is usually declining by 3–6 days of illness. In addition to protein synthesis, there are many unknown functions of ribosomal proteins [27], [28], [29]. For example, L13a is involved in IFN-g-mediated translational inhibition in human monocytes [30]. A better understanding of the relationship between ribosomal proteins and DENV infection and shock syndrome requires additional investigation. The shock-associated genes that we observed have not been noted in other transcriptional profiling studies of children with septic shock [31]. In those studies, shock was associated with down-regulation of genes involved in zinc-related biology and adaptive immunity [31]. This is not surprising, since dengue shock is a very different clinical entity from septic shock [1].

While the commonly observed interferon signature from *in vitro* host-pathogen expression profiling studies is also observed with our meta-analysis of *in vitro* DENV infection, this signature is not consistently observed from *in vivo* patient samples in all studies. Except for CD38, we did not identify many immune-related genes, or genes involved in IFN signaling from DENV infection in the Nicaragua study. This is most likely due to the dynamic nature of acute DENV infection. Unfortunately, accurate defervescence data was not available for all of these study samples collected in 2003–2004, which meant it was not possible to relate the transcriptionl profiles with timing of defervescence. Future longitudinal sampling studies will be needed to examine more closely the dynamic nature of changes in transcriptional profiles over time. From a clinical perspective, our convalescent samples were collected approximately two weeks after symptom onset, as compared to the convalescent samples by Long et al. [9] which were collected at the one month follow-up visit. In our case, the IFN-driven immune response may not have subsided, which would mask the differences in our convalescence comparisons. However, it should be noted that most of our convalescent samples still cluster with the samples collected from healthy volunteers (Figure 1A), indicating that they are more similar to uninfected individuals than to acutely infected individuals. Another limitation of this study is that we did not collect sufficient convalescent samples to make autologous comparisons. This may influence the identification of differentially expressed genes between acute and convalescent samples. The explanation we favor for the difference between our study and the study by Long et al. [9] is that the IFN responsive genes are expressed very early and transiently during acute DENV infection. The Nicaragua study and the earlier Simmons et al. [10] study contained samples that were collected 1–2 days later during the infection cycle than in the Long et al. [9] study, and this difference may well explain the lack of IFN response genes observed in the former studies. The manifestations of DENV infection could also be different between Southeast Asian populations in comparison to the Nicaraguan population that we characterized. Lastly, infection by different circulating DENV serotypes could also contribute to differences in host response.

We noted with interest that Long et al. [9] also observed a significantly higher expression of LTF, DEFA3 and DEFA4 in patient samples with DSS [9]. We found that LTF and DEFA1 were up-regulated in DHF patients but not in DSS patients. Nonetheless, expression of this group of anti-microbial peptides is clearly associated with clinical manifestations more severe than uncomplicated DF. Lactotransferrin (or lactoferrin), cathelicidin (or LL-37) and the defensins are all present in mucosal secretions, are produced at high levels by neutrophils, and have all been shown to have antiviral (especially anti-HIV) activity [32], [33], [34], [35], [36]. Since nothing is known about the role of these molecules in DENV infection, further studies should explore their functions in pathogenesis or anti-DENV immunity.

We also noted that CD14 was up-regulated in DHF patients relative to both DF and DSS patients. This was consistent with the study by de Kruif et al. [37], who found that CD14 was down-regulated in Indonesian children with DSS, relative to DF and DHF cases. CD14 was also down-regulated in DSS patients from Vietnam [10]. CD14 is a monocyte marker and monocytes (as well as macrophages) are known to be the major target cells for DENV infection [38] in patients [39], [40], [41] as well as in mouse models [42]. Further transcriptional profiling efforts focused on this population of cells may reveal interesting mechanisms of pathogenesis.

Accurate gene expression profiling of peripheral blood is challenging because isolating peripheral blood mononuclear cells (PBMCs) by density gradient centrifugation can interfere with biological signatures, especially if the timing between the blood draw and cell isolation is variable [43]. While PAXgene tubes greatly reduce the time between blood draw and RNA stabilization, they introduce abundant globin mRNA that may interfere also with biological signatures [44], [45]. In this study, we used PAXgene tubes in combination with a globin removal system to reduce interference as much as possible. Unlike profiling PBMCs, PAXgene tubes include the transcriptional signatures of reticulocytes, neutrophils and other polymorphonuclear (PMN) cells, hence there are likely to be differences between profiling studies of PBMCs relative to PAXgene samples. For example, the differences in expression of neutrophil-derived anti-microbial peptides would probably not be observed in PBMC studies.

A diagnostic assay that can assist clinicians assess the relative risk of patients progressing towards severe forms of the disease would be a very powerful tool for clinical management. Earlier sampling than was conducted in this study may be required to identify markers for such an assay. Although none of the transcriptional profiling studies that have been conducted so far can claim to have identified genes that have been validated for prognostic potential, it is conceivable that in the near future we can combine datasets from multiple studies through the type of meta-analysis that we have initiated here to identify candidate genes that can then be tested independently in other clinical studies, perhaps at multiple sites. As additional data becomes available, such a dengue pathogenesis assay, based on clinical markers and expression profiles of a few prognostic genes may become closer to reality.

## Supporting Information

Figure S1 Principal Component Analysis of transcriptional profiling data. (A) Plot showing the amount of variance (on the Y-axis) that is explained by each of the principal components (PCA). The results show that the first two principal components (PCA1 and PCA2) contribute the most towards the total variance. (B) Scatterplot of the eigenvector values for principal component 2 for dengue infected individuals. The results show that the values for PCA2 are significantly greater in the DSS patients. (C) Histogram showing the genes identified as contributing towards the top 5% of the positive variance explained by PCA2. (D) Reclustering of samples based on the expression values of 90 genes identified to be the top 5% of genes that are positively correlated with PCA2 (out of N = 1832). (E) Gene Ontology analysis of the 90 genes described above showing the biological processes that are over-represented by these genes.
(0.76 MB PDF)

Table S1 Gene Ontology (GO) analysis of the biological processes and molecular functions that are over-represented by a set of 61 genes that could be related to shock because they contribute towards the top 5% of the positive variance explained by PCA2.
(0.01 MB XLS)

Table S2 Identification of genes that are significantly upregulated in acute DF samples relative to convalescent samples by two-way supervised comparisons using SAM.
(0.10 MB XLS)

Table S3 Identification of genes that are significantly upregulated in acute DHF samples relative to convalescent samples by two-way supervised comparisons using SAM.
(0.07 MB XLS)

Table S4 Identification of genes that are significantly upregulated in acute DSS samples relative to convalescent samples by two-way supervised comparisons using SAM.
(0.11 MB XLS)

Table S5 Identification of genes that are significantly different between patients of different clinical classifications by two-way supervised comparisons using SAM.
(0.03 MB XLS)

Table S6 Gene Ontology (GO) analysis of the biological processes and molecular functions that are overrepresented by genes identified to be differentially expressed between acute DF and DSS samples.
(0.02 MB XLS)

Table S7 Gene Ontology (GO) analysis of the biological processes and molecular functions that are overrepresented by genes identified to be differentially expressed between acute DHF and DSS samples.
(0.01 MB XLS)

Table S8 Identification of a set of “common dengue response” genes for both in vitro and in vivo transcriptional profiling studies through a meta-analysis of 5 separate studies. Genes upregulated by DENV infection are shown in red and genes downregulated are shown in green.
(0.50 MB XLS)

Table S9 Identification of genes that are significantly different from in vivo transcriptional profiling studies that have been conducted previously through a meta-analysis of 3 separate studies. Genes upregulated by DENV infection are shown in red and genes downregulated are shown in green.
(1.66 MB XLS)

Table S10 Identification of genes that are significantly different from in vitro transcriptional profiling studies that have been conducted previously through a meta-analysis of 2 separate studies. Only upregulated genes were found in this analysis.
(0.06 MB XLS)

Table S11 Identification of genes that significantly different between in vitro and in vivo studies by two-way comparisons through meta-analysis. Genes that are relatively upregulated in in vitro studies are shown in red and genes that are relatively upregulated in in vivo studies are shown in green.
(0.30 MB XLS)

Table S12 Identification of genes that are significantly different between severe (DSS) versus non severe (Df and DHF) cases through a meta-analysis of 3 separate studies. Genes upregulated in severe DSS patients are shown in red and genes more highly expressed in non severe DF/DHF patients are shown in green.
(0.05 MB XLS)
