# Supplementary material for: Gene Expression Patterns of Dengue Virus-Infected Children from Nicaragua Reveal a Distinct Signature of Increased Metabolism
Source: PLoS Negl Trop Dis. 2010 Jun 15;4(6):e710. doi: 10.1371/journal.pntd.0000710 (PMC2886038; doi:10.1371/journal.pntd.0000710)
Supplement: Figure S1 — Principal Component Analysis of transcriptional profiling data. (A) Plot showing the amount of variance (on the Y-axis) that is explained by each of the principal components (PCA). The results show that the first two principal components (PCA1 and PCA2) contribute the most towards the total variance. (B) Scatterplot of the eigenvector values for principal component 2 for dengue infected individuals. The results show that the values for PCA2 are significantly greater in the DSS patients. (C) Histogram showing the genes identified as contributing towards the top 5% of the positive variance explained by PCA2. (D) Reclustering of samples based on the expression values of 90 genes identified to be the top 5% of genes that are positively correlated with PCA2 (out of N = 1832). (E) Gene Ontology analysis of the 90 genes described above showing the biological processes that are over-represented by these genes. (0.76 MB PDF) [file pntd.0000710.s001.pdf]

Supplementary Figure 1

A

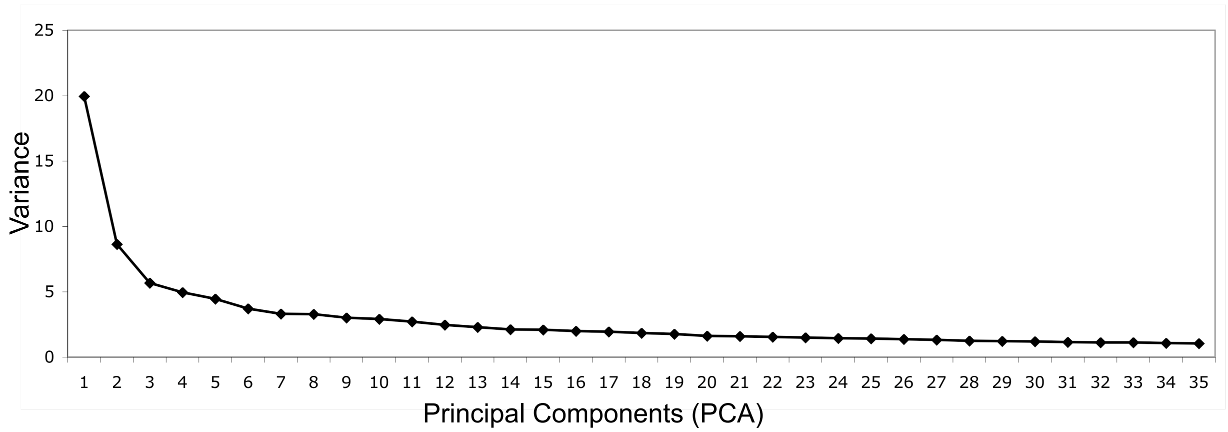

B

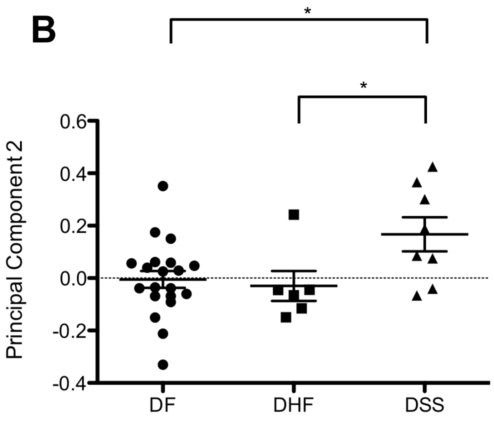

C

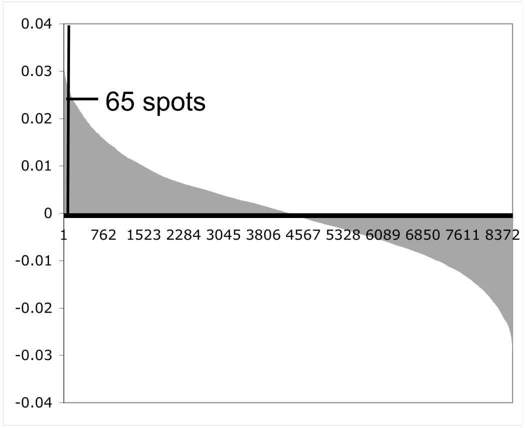

D

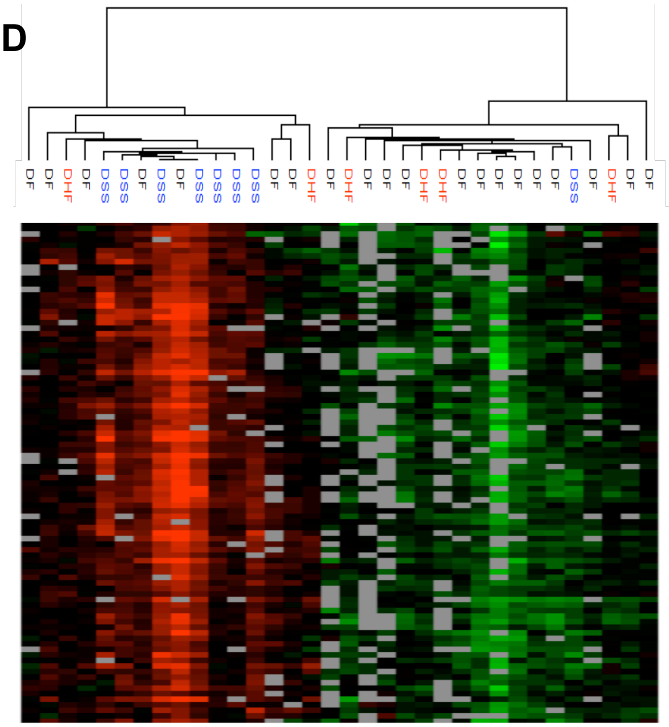

E

Biological Process	(P-value)	Expected	Observed	Gene Symbols
Protein metabolism and modification	3.94E-04	10.76	26	MRPL3, ATAD1, B3GNT2, PSMA6, DPM1, WWP1, UBE2V2, CUL2, RABGGTB, USP1, STK17B, SENP7, NAT13, C15orf15, BZW1, PTPN11, VPS4B, CLK1, PSMC6, TXNDC10, LYPLA1, LRPPRC, SAE2, unassigned
Protein modification	3.18E-02	4.09	13	B3GNT2, DPM1, UBE2V2, RABGGTB, STK17B, NAT13, PTPN11, CLK1, TXNDC10, LYPLA1, LRPPRC, SAE2, unassigned
